# External validation of a United Kingdom primary‐care Cushing's prediction tool in a population of referred Dutch dogs

**DOI:** 10.1111/jvim.16848

**Published:** 2023-09-04

**Authors:** Bart Eduardus Wilhelmus Ruijter, Céline Anne Bik, Imogen Schofield, Stijn Johannes Maria Niessen

**Affiliations:** ^1^ MCD‐AniCura – Internal Medicine, Isolatorweg 45 Amsterdam 1014AS The Netherlands; ^2^ Royal Veterinary College, Hawkshead Lane Hatfield AL9 7TA United Kingdom; ^3^ Royal Veterinary College – Veterinary Clinical Sciences, North Mimms Herts United Kingdom; ^4^ Veterinary Specialist Consultations Hilversum The Netherlands

**Keywords:** adrenal gland, canine, Cushing's syndrome, endocrinology, hyperadrenocorticism, prediction tool, validation

## Abstract

**Background:**

A prediction tool was developed and internally validated to aid the diagnosis of Cushing's syndrome in dogs attending UK primary‐care practices. External validation is an important part of model validation to assess model performance when used in different populations.

**Objectives:**

To assess the original prediction model's transportability, applicability, and diagnostic performance in a secondary‐care practice in the Netherlands.

**Animals:**

Two hundred thirty client‐owned dogs.

**Methods:**

Retrospective observational study. Medical records of dogs under investigation of Cushing's syndrome between 2011 and 2020 were reviewed. Dogs diagnosed with Cushing's syndrome by the attending internists and fulfilling ALIVE criteria were defined as cases, others as non‐cases. All dogs were scored using the aforementioned prediction tool. Dog characteristics and predictor‐outcome effects in development and validation data sets were compared to assess model transportability. Calibration and discrimination were examined to assess model performance.

**Results:**

Eighty of 230 dogs were defined as cases. Significant differences in dog characteristics were found between UK primary‐care and Dutch secondary‐care populations. Not all predictors from the original model were confirmed to be significant predictors in the validation sample. The model systematically overestimated the probability of having Cushing's syndrome (*a* = −1.10, *P* < .001). Calibration slope was 1.35 and discrimination proved excellent (area under the receiver operating curve = 0.83).

**Conclusions and Clinical Importance:**

The prediction model had moderate transportability, excellent discriminatory ability, and overall overestimated probability of having Cushing's syndrome. This study confirms its utility, though emphasizes that ongoing validation efforts of disease prediction tools are a worthwhile effort.

**Abbreviations**
ACTH: adrenocorticotropic hormone
ADH: adrenal‐dependent hypercortisolism
ALP: alkaline phosphatase
AUROC: area under the receiver operating characteristic curve
LDDST: low dose dexamethasone suppression test
NPV: negative predictive value
o‐HDDST: oral high dose dexamethasone suppression test
PDH: pituitary‐dependent hypercortisolism
PPV: positive predictive value
SE: standard error
UCCR: corticoid‐to‐creatinine ratio
USG: urine specific gravity

## 1 INTRODUCTION

Cushing's syndrome is an umbrella term for a range of clinical syndromes that is caused by a chronic excess of glucocorticoid activity, which can be because of a range of endogenous or exogenous steroid hormones.^1^ It is a common endocrine disorder in dogs. Overall prevalence is estimated at 0.17%‐0.28%.^2^, ^3^, ^4^ Spontaneous Cushing's syndrome is caused by an excessive production of glucocorticoids, often leading to a typical case presentation. Common clinical signs include polydipsia, polyuria, polyphagia, panting, abdominal enlargement, hepatomegaly, dermatological changes, and muscle atrophy. Frequently observed clinicopathological abnormalities include a stress leukogram, increased serum alkaline phosphatase activity (ALP), and hypercholesterolaemia.^5^, ^6^

Multiple adrenal function tests and differentiating tests are described for diagnostic purposes, including urine corticoid‐to‐creatinine ratio (UCCR) with or without suppression using oral dexamethasone, low dose dexamethasone suppression test (LDDST) on the basis of blood cortisol, and adrenocorticotropic hormone (ACTH) stimulation test also on the basis of blood cortisol measurement. However, none of these tests are perfect, as they can be time‐consuming, costly, and both false‐positive and false‐negative results are common.^6^, ^7^, ^8^, ^9^, ^10^, ^11^, ^12^ Recently, a prediction tool was developed and internally validated to aid the diagnosis of spontaneous Cushing's syndrome in dogs.^13^, ^14^ This model demonstrated a good predictive performance in dogs attending UK primary‐care practices, using neuter status, age, breed, polydipsia, vomiting, potbelly/hepatomegaly, alopecia, pruritus, urine specific gravity (USG), and serum ALP as predictor variables.

A prediction model can be validated internally and externally. With internal validation, the model is tested in patients who belong to the original population and indicates how the model would likely perform in a very similar population. With external validation, the model is tested in patients belonging to another population. External validation is an important part of model validation, as different population characteristics could have a major influence on the performance of a prediction model.^15^, ^16^ These variations in population characteristics have been categorized as temporal, geographic, and domain differences.^17^, ^18^ With temporal validation, the same study is performed in a similar population from a later time period. With geographic validation, the model performance is tested by varying the location of the population (eg, primary‐care practices in the UK vs primary‐care practices in the Netherlands). Domain validation can be performed when one suspects differences in patient groups (eg, primary‐care vs secondary‐care practice). Disease prevalence and characteristics can vary markedly between primary‐care and secondary‐care or tertiary‐care practices in both human and veterinary medicine.^19^, ^20^, ^21^ The aim of this study was to assess the aforementioned prediction model's transportability, applicability, and diagnostic performance in a secondary‐care practice in the Netherlands.

## 2 MATERIALS AND METHODS

### 2.1 Case selection

All electronic medical records of the Internal Medicine service of small animal referral clinic “Amsterdam Medisch Centrum voor Dieren” containing results of UCCR's in combination with an oral high dose dexamethasone suppression test (most commonly used in the Netherlands and at the investigational hospital) between January 2011 and December 2020 were reviewed. The use of UCCR and oral high dose dexamethasone suppression test (o‐HDDST) has previously been validated^9^, ^22^, ^23^ and involved the collection of a morning urine sample by the owner on 3 consecutive days. After collection of the second urine sample, the owner administered 3 oral doses of dexamethasone (0.1 mg/kg/dose) at 8‐hour intervals. UCCR was measured in all 3 morning urine samples. In an animal with appropriate signalment and suggestive clinical signs hypercortisolism was suspected if the average of the first 2 UCCRs was ≥10 × 10^−6^; this cut‐off was previously established by the University of Utrecht Veterinary Laboratory.^9^ If the third UCCR was <50% of the mean of the first 2 samples, this was considered suggestive of pituitary‐dependent hypercortisolism (PDH). According to the described validation of the methodology, a decrease >50% was considered to be suggestive of pituitary‐dependent (but dexamethasone‐resistant) hypercortisolism, ectopic ACTH excess, or ACTH independence (eg, adrenal‐dependent hypercortisolism [ADH]). The same laboratory and assay were used for UCCR measurement as in the validation publications.

Dogs were included as cases (ie, having Cushing's syndrome) if this was the final conclusion of the attending internist based on a combination of medical history, clinical signs, physical examination, routine laboratory investigations, endocrine tests (UCCR and o‐HDDST), and diagnostic imaging (abdominal ultrasound ± computed tomography). Cases were excluded if a subsequent revision of the diagnosis was made in the medical record. Dogs were included as non‐cases (ie, not having Cushing's syndrome) if the attending internist considered Cushing's syndrome and subsequently ruled out this diagnosis based on normal UCCRs and o‐HDDST in combination with 1 or more of the following: medical history, clinical signs, physical examination, routine laboratory investigations, other endocrine tests, and diagnostic imaging. A definite alternative diagnosis was not required for non‐cases.

All cases and non‐cases were subsequently independently reviewed by the primary and last author and verified to be compliant (cases) or not (non‐cases) with ALIVE criteria for diagnosis of Cushing's syndrome.^24^, ^25^ Those not fulfilling ALIVE criteria were excluded. Current ALIVE criteria for PDH and ADH are shown in Table 1.

**TABLE 1:** Current ALIVE criteria for diagnosis of pituitary‐dependent hypercortisolism and adrenal‐dependent hypercortisolism.

Pituitary‐dependent hypercortisolism criteria	Accepted ways to fulfill criteria
Identification of a set of clinical features attributable to Cushing's syndrome including.	Supportive history, physical examination findings and clinicopathologic test results
Demonstration of an excess of cortisol through dynamic testing of pituitary‐adrenal function	Dexamethasone suppression test based on blood OR Dexamethasone suppression test based on urine OR ACTH stimulation test
ACTH‐dependence originating from the pituitary is proven through at least 1 clear differentiation test result	Characteristic suppression of a LDDST using blood OR Characteristic suppression of a HDDST using blood OR Characteristic suppression of a HDDST combined with UCCR measurement OR Absence of suppressed endogenous ACTH concentration OR Absence of an ultrasound examination characteristic of a glucocorticoid‐secreting adrenal tumor using ALIVE methodology OR Characteristic changes of pituitary morphology on CT or MRI using ALIVE methodology.

Adrenal‐dependent hypercortisolism criteria	Accepted ways to fulfill criteria
Identification of a set of clinical features attributable to Cushing's syndrome including.	Supportive history, physical examination findings and clinicopathologic test results
Demonstration of an excess of cortisol through dynamic testing of pituitary‐adrenal function	Dexamethasone suppression test based on blood OR Dexamethasone suppression test based on urine OR ACTH stimulation test
ACTH‐independence confirmed by at least 1 clear differentiation test result	Suppressed endogenous ACTH concentration OR Ultrasound examination characteristic of a glucocorticoid‐secreting adrenal tumor using ALIVE criteria OR Characteristic changes of adrenal morphology on ultrasound, CT or MRI using ALIVE methodology.

*Note*: Fulfillment of all 3 criteria is required for diagnosis.

### 2.2 Prediction tool

The published Cushing's Prediction Tool (Table 2) was used as described in the aforementioned study.^14^ Information regarding clinical signs and laboratory results within 1 week before and 1 week after the point of first assessment at the internal medicine service were used for scoring purposes. If a specific clinical sign was not mentioned in the medical record, it was considered to be absent.

**TABLE 2:** Prediction tool to calculate the likelihood of a dog having Cushing's syndrome.

	Category	Points
Dog demography		
Neuter status	Female‐entire	0
	Female‐neutered	−1
	Male‐entire	−1
	Male‐neutered	−1
Current age (years)	<7	0
	≥7	1
Breed	Bichon frise	2
	Border terrier	1
	Labrador retriever	−3
	Schnauzer	−2
	West Highland white terrier	−3
	Other breed or crossbreed	0
Presenting clinical signs		
Polydipsia	Yes	2
	No	0
Vomiting	Yes	−2
	No	0
Potbelly/hepatomegaly	Yes	3
	No	0
Alopecia	Yes	2
	No	0
Pruritus	Yes	−2
	No	0
Laboratory factors		
Urine specific gravity	Dilute (≤1.020)	0
	Not dilute (>1.020)	−2
	Not recorded	−1
Serum ALP	Elevated	0
	Not elevated	−3
	Not recorded	0

*Note*: To calculate the predicted likelihood of an individual dog having Cushing's syndrome, one has to add together the points that correspond to the category for each predictor and match the final score to the predicted likelihood as published by Schofield et al.^14^ This way, a likelihood between 0% (score −13) and 96% (score 10) can be predicted.

### 2.3 Additional laboratory factors and comorbidities

In addition to the baseline characteristics used as predictors in the prediction tool, the presence or absence of lymphopenia, hypercholesterolemia, persistent proteinuria using urine protein‐to‐creatinine ratio, urinary tract infection using culture, uroliths using imaging, vacuolar hepatopathy using cytology or histopathology, diabetes mellitus, systemic hypertension, thromboembolic disease (considered absent if not reported), and gallbladder sludge or mucocele using ultrasound were noted for every case.

### 2.4 Statistical analysis

Statistical analysis was performed using R (R version 4.2.1, and RStudio v2022.02.3+492; packages mosaic, car, Hmisc, Epi, PredictABEL, and fmsb). Baseline characteristics were expressed as n and % for categorical variables. Subgroup differences in baseline characteristics were examined using Wilcoxon's rank sum test for non‐normally distributed numeric variables and Fisher's exact test for categorical variables.

The first step in the external validation of the diagnostic prediction tool was to subjectively qualify the level of relatedness between the case mix of the development and validation sample (model transportability). This is an important step, as it helps to differentiate between reproducibility and transportability. Reproducibility refers to a model's capacity to produce accurate predictions in a new sample that is very similar to the development population, whereas transportability refers to the ability to produce accurate predictions in a different population. To assess model transportability, one would ideally see a low to moderate degree of relatedness. If the development and validation sample appear to be (almost) identical, the external validation study could actually reflect the model's reproducibility.^26^, ^27^ Using the summary measures percentages, median and range, the distribution of the dog characteristics was compared. This included the predictors in the validated model and outcome occurrence. Furthermore, the extent to which the development and validation samples share common predictor effects was evaluated by refitting the original logistic regression model in the validation sample. The estimated regression coefficients and corresponding SEs were compared to evaluate the heterogeneity in the predictor‐outcome associations.

The second step of external validation involved examining the calibration and discrimination to assess the model's performance in the new validation sample. Calibration measures the agreement between observed and predicted outcomes. Calibration‐in‐the‐large was given as the intercept term *a* from the recalibration model logit(y) = *a* + *b* × logit(ŷ), in which the logit(y) is the natural logarithm of the observed odds of being diagnosed with Cushing's syndrome and logit(ŷ) the natural logarithm of the predicted odds of being diagnosed with Cushing's syndrome. Ideally, the intercept term *a* should equal 0. If *a* < 0, this indicates the model overestimates the odds, whereas *a* > 0 indicates underestimation. The calibration slope was estimated as b from the same recalibration model. Ideally, the calibration slope b equals 1. If 0 < *b* < 1, this often indicates predictions vary too much (ie, too low with low predicted probability, too high with high predicted probability), whereas *b* > 1 implies the opposite. Discrimination was evaluated calculating the c‐statistic with 95% confidence intervals (CI), corresponding to the area under the receiver operating characteristic curve (AUROC) for the outcome diagnosis Cushing's syndrome. This reflects whether individual dogs with Cushing's syndrome receive a higher predicted probability than those without. For c‐statistic, 0.5‐0.7 was interpreted as poor performance, 0.7‐0.8 as acceptable performance, 0.8‐0.9 as excellent performance, and >0.9 as outstanding performance.^28^ Significance was set at *P* < .05 for all analyses.

## 3 RESULTS

### 3.1 Descriptive statistics

In total, 242 medical records were identified. Twelve cases were excluded because they could not be assessed to be case or non‐case using the ALIVE criteria. Of 230 dogs, 26 (11.3%) were female‐entire, 107 (46.5%) female‐neutered, 38 (16.5%) male‐entire, and 59 (25.7%) male‐neutered. Median age was 10.2 years and there was no significant difference between cases and non‐cases (10.5 years [range 5.0‐15.7] and 10.1 years [range 2.1‐16.0], respectively; *P* = .30). The most commonly observed breeds in the study sample included crossbreed (n = 44 [19.1%]), Jack Russel terrier (n = 15 [6.5%]), Beagle (n = 11 [4.8%]), Dachshund (n = 10 [4.3%]), Cairn terrier (n = 9 [3.9%]), Maltese (n = 9 [3.9%]), French bulldog (n = 8 [3.5%]), Shih Tzu (n = 7 [3.0%]), Labrador retriever (n = 6 [2.6%]), Poodle (n = 5 [2.2%]), and Yorkshire terrier (n = 5 [2.2%]).

Eighty dogs (34.8%) were defined as cases with Cushing's syndrome using ALIVE criteria. In 65/80 dogs (81.3%) the disease was considered pituitary‐dependent, in 12/80 (15%) adrenal‐dependent, and in 3/80 (3.8%) sub‐type could not be specified. Final diagnosis was available for 125/150 non‐cases (83.3%; Table 3). Baseline characteristics are presented in Table 4, stratified for cases and non‐cases.

**TABLE 3:** Final diagnosis recorded in the medical records for non‐cases (n = 150).

Disease category	Non‐cases (%)	Final diagnosis
Cardiorespiratory	6 (4.0)	Brachycephalic obstructive syndrome (1), chronic bronchitis (1), dilated cardiomyopathy (1), myxomatous mitral valve disease (1), pulmonic stenosis (1), tracheal collapse (1)
Dermatological	2 (1.3)	Atopic dermatitis (2)
Endocrine	23 (15.3)	Central diabetes insipidus (6), diabetes mellitus (12), idiopathic hypercalcemia (1), pheochromocytoma (1), primary hyperparathyroidism (1), primary hypothyroidism (2)
Gastrointestinal	20 (13.3)	Acute gastroenteritis (1), chronic enteropathy (18), periodontal disease (1)
Hepatobiliary	4 (2.7)	Cholelithiasis (2), hepatic amyloidosis (1), reactive hepatitis (1)
Miscellaneous	14 (9.3)	Iatrogenic Cushing's (1), overfeeding (5), postprandial hyperlipemia (1), side effect phenobarbital (2), transient polydipsia (3), underfeeding (2)
Neoplastic	10 (0.7)	Apocrine gland anal sac adenocarcinoma (2), brain tumor (1), hepatic mass (1), mammary gland tumor (1), multicentric lymphoma (1), pulmonary mass (1), splenic haemangioma (1), splenic haemangiosarcoma (1), urinary bladder transitional cell carcinoma (1)
Neurological	12 (8.0)	Cerebrovascular event (1), psychogenic polydipsia (10), vestibular geriatric syndrome (1)
Ocular	5 (3.3)	Sudden acquired retinal degeneration syndrome (5)
Orthopedic	1 (0.7)	Osteoarthritis (1)
Renal	18 (12.0)	Chronic kidney disease (10), focal and segmental glomerulosclerosis (1), primary nephrogenic diabetes insipidus (1), protein losing nephropathy (1), pyelonephritis (3), renal glucosuria (2)
Urogenital	10 (0.7)	Urinary bladder polyp (1), silent heat (1), urethral sphincter mechanism incompetence (3), urinary tract infection (4), urolithiasis (1)
No final diagnosis	25 (16.7)	Adrenal mass (4), suspected alopecia X (1), suspected chronic enteropathy (6), suspected chronic lymphatic leukemia (1), suspected Cushing's, lost on follow‐up (1), suspected intracranial disease (2), suspected dermatological disorder (1), psychogenic polydipsia vs primary nephrogenic diabetes insipidus (9)

**TABLE 4:** Baseline characteristics and Fisher's exact association stratified for cases and non‐cases.

	Category	Cases (%)	Non‐cases (%)	*P* value
Dog demography				
Neuter status	Female‐entire	9 (11.3)	17 (11.3)	.01*
	Female‐neutered	29 (36.3)	78 (52.0)	
	Male‐entire	22 (27.5)	16 (10.7)	
	Male‐neutered	20 (25.0)	39 (26.0)	
Current age (years)	<7	7 (8.8)	25 (16.7)	.21
	7‐11	39 (48.8)	60 (40.0)	
	≥11	34 (42.5)	65 (43.3)	
Breed	Beagle	6 (7.5)	5 (3.3)	.54
	Bichon frise	1 (1.3)	0 (0.0)	
	Border terrier	0 (0.0)	2 (1.3)	
	Cairn terrier	3 (3.8)	6 (4.0)	
	Crossbreed	16 (20.0)	28 (18.7)	
	Dachshund	2 (2.5)	8 (5.3)	
	French bulldog	6 (7.5)	2 (1.3)	
	Jack Russell terrier	7 (8.8)	8 (5.3)	
	Labrador retriever	1 (1.3)	5 (3.3)	
	Maltese	1 (1.3)	8 (5.3)	
	Other purebreed	31 (38.8)	63 (42.0)	
	Poodle	2 (2.5)	3 (2.0)	
	Schnauzer	0 (0.0)	1 (0.7)	
	Shih Tzu	3 (3.8)	4 (2.7)	
	West Highland white terrier	0 (0.0)	3 (2.0)	
	Yorkshire terrier	1 (1.3)	4 (2.7)	
Presenting clinical signs				
Polydipsia	Yes	75 (93.8)	124 (82.7)	.03*
	No	5 (6.3)	20 (13.3)	
Vomiting	Yes	3 (3.8)	20 (13.3)	.02*
	No	77 (96.3)	130 (86.7)	
Potbelly/hepatomegaly	Yes	57 (71.3)	64 (42.7)	<.001*
	No	23 (28.8)	86 (57.3)	
Alopecia	Yes	44 (55.0)	27 (18.0)	<.001*
	No	36 (45.0)	123 (82.0)	
Pruritus	Yes	2 (2.5)	9 (6.0)	.34
	No	78 (97.5)	142 (94.0)	
Laboratory factors				
Urine specific gravity	Dilute (≤1.020)	37 (46.3)	54 (36.0)	.07
	Not dilute (>1.020)	4 (5.0)	21 (14.0)	
	Not recorded	39 (48.8)	75 (50.0)	
Serum ALP	Elevated	64 (80.0)	66 (44.0)	<.001*
	Not elevated	7 (8.8)	66 (44.0)	
	Not recorded	9 (11.3)	18 (12.0)	
Lymphocyte count	Decreased	13 (16.3)	14 (9.3)	.02*
	Not decreased	26 (32.5)	76 (50.7)	
	Not recorded	41 (51.3)	60 (40.0)	
Serum cholesterol	Elevated	8 (10.0)	7 (4.7)	.02*
	Not elevated	9 (11.3)	38 (25.3)	
	Not recorded	63 (78.8)	105 (70.0)	
Urine protein‐to‐creatinine ratio	Persistent proteinuria	6 (7.5)	11 (7.3)	.31
	No persistent proteinuria	5 (6.3)	17 (11.3)	
	Not recorded	69 (86.3)	122 (81.3)	
Urinary tract infection	Yes	7 (8.8)	11 (7.3)	.09
	No	11 (13.8)	45 (30.0)	
	Not recorded	62 (77.5)	94 (62.7)	
Urolithiasis	Yes	1 (1.3)	6 (4.0)	.23
	No	70 (87.5)	127 (84.7)	
	Not recorded	9 (11.3)	17 (11.3)	
Vacuolar hepatopathy	Yes	32 (40.0)	42 (28.0)	.14
	No	4 (5.0)	12 (8.0)	
	Not recorded	44 (55.0)	96 (64.0)	
Diabetes mellitus	Yes	0 (0.0)	12 (8.0)	.007*
	No	71 (88.8)	130 (86.7)	
	Not recorded	9 (11.3)	8 (5.3)	
Thromboembolism	Yes	2	1	.28
	No	78	149	
Gall bladder content	Abnormal	10 (12.5)	6 (4.0)	.02*
	Normal	60 (75.0)	122 (81.3)	
	Not recorded	10 (12.5)	22 (14.7)	

* *P* value <.05 implies a significant difference in baseline characteristics of cases and non‐cases.

Sex distribution in cases was significantly different from that in non‐cases (*P* = .01): 29/80 (36.3%) cases were female‐neutered vs 78/150 (52.0%) non‐cases, whereas 22/80 (27.5%) cases were entire males vs 16/150 (10.7%) non‐cases. No statistically significant differences in age, breed, and pruritus were found between cases and non‐cases. Polydipsia, potbelly, and alopecia were more commonly present in cases compared to non‐cases (93.8% vs 82.7%, *P* = .03; 71.3% vs 42.7%, *P* < .001; 55% vs 18.0%, *P* < .001, respectively). Vomiting was reported less frequently in cases than non‐cases (3.8% vs 13.3%, *P* = .02). Serum ALP was more commonly elevated in dogs diagnosed with Cushing's syndrome than in those without (80.0% vs 44.0%, *P* < .001).

Descriptive statistics of additional laboratory variables and comorbidities are presented in Table 4. Systemic blood pressure was excluded from analysis as it was recorded rarely (0/80 cases, 6/150 non‐cases). Lymphopenia was noted more often in cases (13/39 [33.3%] vs 14/90 [15.6%], *P* = .02). Hypercholesterolemia was noted more frequently in cases than non‐cases (8/17 [47.1%] vs 7/45 [15.6%], *P* = .02). Diabetes mellitus was observed less in cases than non‐cases (0/71 [0%] vs 12/142 [8.5%], *P* = .007). None of the non‐cases diagnosed with diabetes mellitus had evidence of insulin resistance. Finally, abnormal gall bladder content was reported more frequently in cases than non‐cases (10/70 [14.3%] vs 6/128 [4.7%], *P* = .02). Abnormal gall bladder content was reported as sludge in 7 cases and 5 non‐cases, and as mucocele in 3 cases and 1 non‐case.

### 3.2 Model transportability

Several significant differences were found in the baseline characteristics of the development data set and the external validation data set (Table 5). Refitting the originally reported model in the current validation data set resulted in the regression coefficients and SEs presented in Table 6. The regression coefficients and standard errors of the original model are shown as a comparison. From the predictors of the original model, the regression coefficients of male‐entire, vomiting, alopecia, USG not dilute, USG not recorded and serum ALP not elevated demonstrated a *P* < .05. The *P* value of the regression coefficient of polydipsia and potbelly proved <.10. All other regression coefficients demonstrated a *P* > .10.

**TABLE 5:** Comparison of baseline characteristics of development data set (primary‐care practice; n = 939) and external validation set (secondary‐care practice; n = 230) and Fisher's exact association.

	Category	Development	External validation	*P* value
Incidence Cushing's syndrome (%)		42.4	34.8	.04*
Dog demography				
Neuter status (%)	Female‐entire	10.3	11.3	.63
	Female‐neutered	41.5	46.5	.18
	Male‐entire	12.1	16.5	.08
	Male‐neutered	36.0	25.7	.003*
Current age (%)	<7	13.2	13.9	.04*
	7‐11	43.6	43.0	.94
	≥11	43.2	43.0	1.0
Breed (%)	Bichon frise	6.0	0.4	<.001*
	Border terrier	3.6	0.9	.03*
	Crossbreed	21.7	19.1	.42
	Jack Russell terrier	8.3	6.5	.42
	Labrador retriever	4.8	2.6	.21
	Other purebreed	36.0	66.1	<.001*
	Schnauzer	3.2	0.4	.02*
	Staffordshire bull terrier	5.9	0.4	<.001*
	West Highland white terrier	6.3	1.3	.002*
	Yorkshire terrier	4.3	2.2	.18
Presenting clinical signs				
Polydipsia (%)	Yes	57.5	86.5	<.001*
	No	42.5	13.5	
Vomiting (%)	Yes	8.3	10.0	.43
	No	91.7	90.0	
Potbelly/hepatomegaly (%)	Yes	33.3	52.6	<.001*
	No	66.7	47.4	
Alopecia (%)	Yes	21.2	30.9	.002*
	No	78.8	69.1	
Pruritus (%)	Yes	6.4	4.8	.44
	No	93.6	95.2	
Laboratory factors				
Urine specific gravity (%)	Dilute (≤1.020)	24.2	39.6	<.001*
	Not dilute (>1.020)	16.0	10.9	.06
	Not recorded	59.8	49.6	.006*
Serum ALP (%)	Elevated	50.5	56.5	.11
	Not elevated	7.3	31.7	<.001*
	Not recorded	42.2	11.7	<.001*

* *P* value <.05 implies a significant difference in baseline characteristics of development data set and external validation set.

**TABLE 6:** Estimated regression coefficients and corresponding standard errors (SE) for Cushing's syndrome prediction model in development data set (primary‐care practice; cases, n = 398; non‐cases, n = 541) and external validation data set (secondary‐care practice; cases, n = 80; non‐cases, n = 150).

Predictor	Category	*r*_dev_	SE_dev_	*r*_val_	SE_val_	*P* value
Constant (model intercept)		−0.49	0.38	−1.31	1.18	.27
Neuter status	Female‐entire	Baseline		Baseline		
	Female‐neutered	−0.64	0.27	−0.20	0.61	.75
	Male‐entire	−0.34	0.32	1.44	0.72	.04*
	Male‐neutered	−0.60	0.27	0.04	0.65	.95
Current age (years)	<7	Baseline		Baseline		
	7 to <11	0.64	0.26	0.47	0.69	.50
	≥11	0.58	0.27	0.05	0.71	.95
Breed	Crossbreed	Baseline		Baseline		
	Bichon frise	0.68	0.34	15.81	3956.18	1.0
	Border terrier	0.61	0.44	−19.45	2315.47	.99
	Jack Russell terrier	0.11	0.30	0.81	0.93	.38
	Labrador retriever	−1.37	0.49	−0.97	1.34	.47
	Other purebred	−0.04	0.20	−0.12	0.50	.81
	Schnauzer	−1.03	0.53	−14.67	3956.18	1.0
	Staffordshire terrier	0.05	0.35	−11.97	3956.18	1.0
	West Highland white terrier	−1.18	0.37	−16.54	1835.12	.99
	Yorkshire terrier	0.09	0.40	−1.15	1.47	.43
Polydipsia	Yes	0.87	0.16	1.16	0.65	.07
	No	Baseline		Baseline		
Vomiting	Yes	−0.76	0.31	−2.16	0.90	.02*
	No	Baseline		Baseline		
Potbelly/hepatomegaly	Yes	1.11	0.17	0.74	0.41	.07
	No	Baseline		Baseline		
Alopecia	Yes	0.94	0.20	2.49	0.48	<.001*
	No	Baseline		Baseline		
Pruritus	Yes	−0.88	0.35	−1.85	1.46	.20
	No	Baseline		Baseline		
Urine specific gravity	Dilute (≤1.020)	Baseline		Baseline		
	Not dilute (>1.020)	−0.85	0.26	−2.02	0.78	.01*
	Not recorded	−0.43	0.20	−1.61	0.48	<.001*
Serum ALP	Elevated	Baseline		Baseline		
	Not elevated	−1.46	0.35	−2.96	0.61	<.001*
	Not recorded	−0.16	0.16	−0.85	0.62	.17

* *P* value <.05 implies a significant predictor‐outcome association after refitting the original logistic regression model in the external validation data set.

Abbreviations: r_dev_, estimated regression coefficient in development data set; r_val_, estimated regression coefficient in external validation data set; SE_dev_, standard error in development data set; SE_val_, SE in external validation data set.

### 3.3 Model performance

**FIGURE 1:**
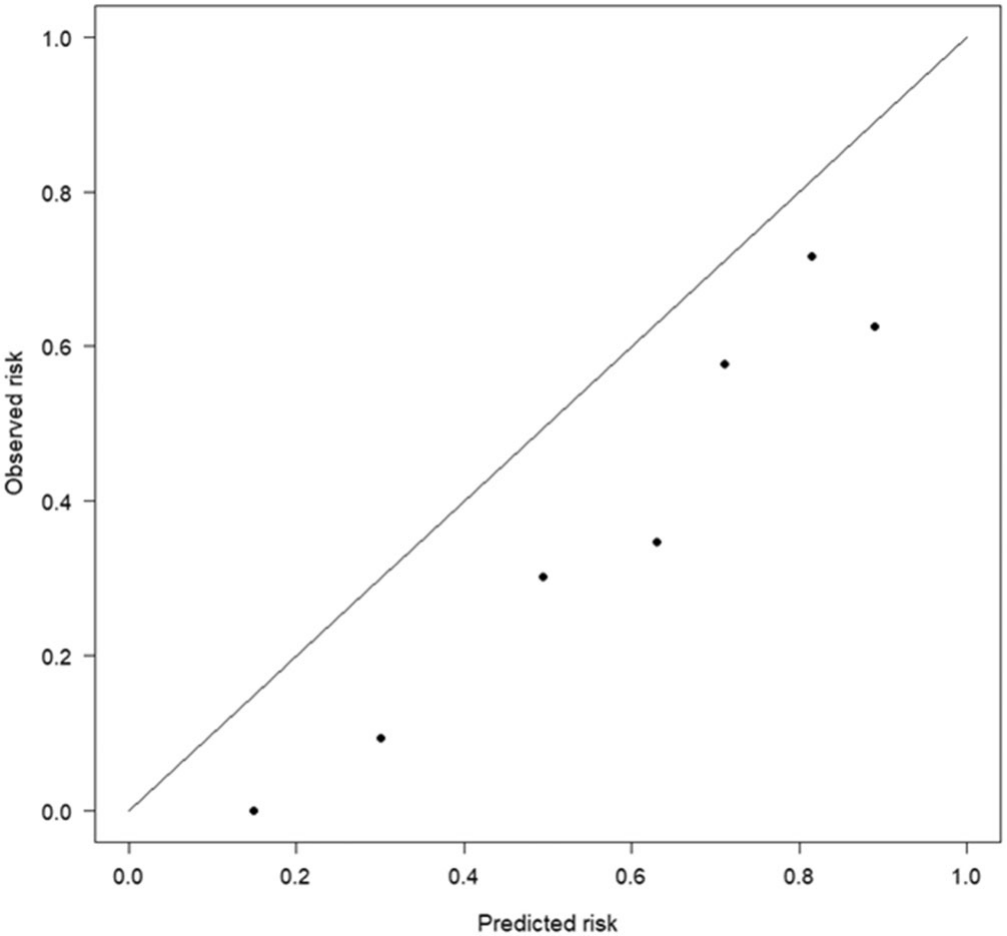
Calibration plot for the outcome diagnosis of Cushing's syndrome. The plot shows the mean observed proportions of dogs with a diagnosis of Cushing's compared to the mean predicted probabilities, by deciles of predictions. The 45° line denotes perfect calibration.

The clinical prediction model systematically overestimated the probability of having Cushing's syndrome (*a* = −1.10 [95% CI: −1.49 to −0.71], *P* < .001). The calibration slope was 1.35 (95% CI: 0.97 to 1.73, *P* < .001; Figure 1). The Hosmer‐Lemeshow test was significant (*P* < .001), indicating that the observed probabilities (expressed as the natural logarithm of the odds) differed significantly from the predicted probabilities.

**FIGURE 2:**
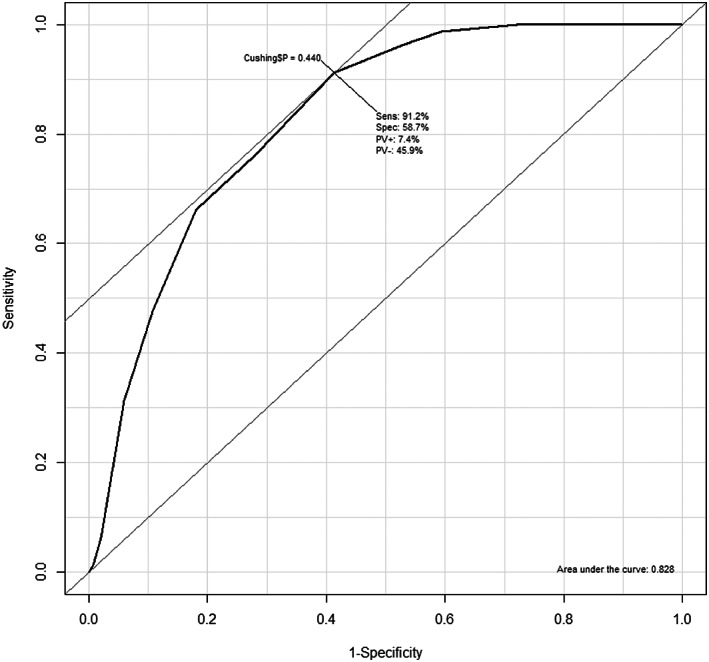
Receiver operating characteristic curve for the outcome diagnosis of Cushing's syndrome.

The discrimination of the model in the current dataset was excellent (c‐statistic = 0.83; Figure 2). When setting the threshold of the prediction tool at 2 for the total prediction score (ie, dogs with prediction tool end score ≥2 are predicted as cases and <2 are predicted as non‐cases), sensitivity was 91% and specificity 59%. This resulted in a negative predictive value (NPV) of 92% and positive predictive value (PPV) of 54% for diagnosis of Cushing's syndrome in this group of referred Dutch dogs. Decreasing the threshold for the total prediction score to ≥0 increased the NPV to 99% (sensitivity 99%, specificity 41%, PPV 47%).

## 4 DISCUSSION

This study showed several significant differences in dog characteristics between development UK primary‐care dogs and the external validation Dutch secondary‐care study sample. Moreover, comparison of the estimated regression coefficients and corresponding standard errors revealed substantial heterogeneity in the predictor‐outcome associations. These findings imply moderate transportability of the model, which means that the Dutch dogs differ from those in the original UK study, supporting that external validation was performed in the current study.

There are several explanations for the moderate transportability of the model (ie, different case mix). Geographical differences could have resulted in the finding that breeds used as predictors in the prediction model were uncommon in our study cohort. One explanation for this could be a difference in breed‐associated risk between the 2 countries, as breeding practices might lead to significant genetic differences and disease predisposition.^29^, ^30^ However, it might also be explained by different breed popularities in the United Kingdom and the Netherlands.

Calibration of the model detected a discrepancy between the predicted and observed odds of being diagnosed with Cushing's syndrome, with the model overestimating the probability of having Cushing's syndrome in the Dutch dogs. Some underfitting of the model was detected, implying less variation in the predictive chance of being diagnosed with Cushing's syndrome (high predictions were too low, whereas low predictions were too high). On the other hand, discriminating ability of the model (ie, can the model distinguish cases from non‐cases) was found to be excellent given an AUROC of 0.83. This was fairly similar to the AUROC of the prediction tool in its development study sample. Overall, this implies that the model showed good performance in this group of dogs used for external validation.

That the current study tested the model's performance in dogs that were presented to a referral hospital instead of a primary‐care practice could explain why the dogs diagnosed with Cushing's syndrome in the present study had higher frequency of clinical signs associated with the condition. This could indicate that dogs diagnosed with Cushing's syndrome in a secondary‐care practice show a more prominent clinical picture compared to those diagnosed in primary‐care practice. Another explanation could be that the attending internists were more likely to recognize or report these clinical signs. Additionally, the dogs with and without Cushing's syndrome were more similar to each other in the current study than those in the UK primary‐care caseload. Dogs without an overly clear clinical picture of Cushing's syndrome but still with some of the suggestive clinical signs and laboratory variables might be referred more often. In addition, veterinarians specialized in internal medicine within secondary‐care practice might be more familiar with and/or confident in assessing the clinical picture of Cushing's syndrome. Indeed, polydipsia, potbelly/hepatomegaly, and alopecia were noted more often in non‐cases of the external validation group than non‐cases of the development group. Such differences could explain why the model overestimates the probability of having Cushing's syndrome in the current cohort's non‐cases.

In the current study, only a few of the original model's predictors were found to be significant in the external validation sample (male‐entire, vomiting, alopecia, not diluted or not recorded USG, and not elevated serum ALP). This explains the substantial heterogeneity in the predictor‐outcome associations. A possible explanation for this is that the other predictors were selected for the original prediction tool because of overfitting. This phenomenon seems to be greatest for the selected breeds. Another factor to consider is the definition of the cases and non‐cases in this study vs the original study reporting the development and validation of the Cushing's Prediction Tool. Internationally agreed ALIVE criteria were used as recommended by the 2 largest veterinary endocrinology societies in the current study, whereas the ALIVE criteria were not literally applied to the original 1.

The current study shows a different behavior of the original tool when applied in a sample of a different population. This emphasizes the ongoing need to further externally validate the tool in different populations and settings. Further external validation of the prediction tool would particularly be beneficial within other primary‐care populations, other geographical domains and clinic‐settings. In addition, several possibilities exist to improve the model's performance for use within secondary‐care practice in the Netherlands, without creating a new model completely. As a first possible step, the intercept could be updated for a better calibration.^26^, ^31^ Second, to improve the model's discriminating ability, removing predictors from the original model could be considered, since several of the original predictors were not associated with outcome in our study (eg, breeds). Adding new predictor variables to the original model could be a third step. In the Dutch dogs, lymphopenia, hypercholesterolemia, and abnormal gall bladder content (sludge, mucocele) proved more common in cases than non‐cases. These variables were selected because of the evidence in the literature to indicate their discriminatory ability between dogs with and without Cushing's syndrome.^32^, ^33^, ^34^ These adjustments were not part of the scope of this study. A new prediction model was not constructed using the Dutch data set, as the number of cases and non‐cases was relatively low. This could lead to overfitting, rendering the improved or new model not useful for future predictions.^35^

The retrospective nature of the current study represents an obvious limitation. Laboratory findings were for instance not always available. Although the prediction model was developed to also be used when laboratory findings were not available, it might have shown a better performance with more data available. Moreover, clinical signs not mentioned in the medical record were considered to be absent. However, it is possible that they were present but not recorded, leading to incorrect scores. The authors chose to use the internationally agreed ALIVE criteria for diagnosis of PDH and ADH. The veterinary endocrinology communities actively promote the use of ALIVE definitions since they foster uniformity and comparability of data.^36^ The results of this study should therefore be seen in the light of these specific disease definitions. Results could therefore also have been different when differing definitions were used, such as in the original British study. The same applies to the use of UCCR in combination with o‐HDDST for the diagnosis and differentiation of Cushing's syndrome. This test is popular in the Netherlands, yet not commonly used internationally.^37^ A drawback of the prediction model itself is that it uses predictors that are also part of the reference standard for diagnosis (ie, the opinion of the attending veterinarian, based on a combination of medical history, clinical signs, physical examination, routine laboratory investigations, endocrine tests, and diagnostic imaging). This is known as incorporation bias, and it can lead to overestimation of the diagnostic accuracy.^38^ Finally, an important limitation to note is the sample size. Ideally, a large‐scale dataset is used for validation purposes to prevent imprecise predictive performance estimates. To minimize this effect, statistical methods were used to account for potential overestimation of model performance because of model fitting on the same dataset. This correction ensures a more accurate estimation of the model's predictive ability and helps mitigate any potential bias introduced by the relatively low numbers of cases.

In conclusion, this external validation study showed moderate transportability of a Cushing's syndrome prediction model developed in the UK in a group of dogs presented to a secondary‐care practice in the Netherlands. The model had excellent discriminatory ability. Overall, the tool did overestimate the probability of having Cushing's syndrome. Despite its limitations, the tool could still prove useful in its current form. Using a total prediction score cut‐off of 0 (ie, score < 0 predicts the dog does not have Cushing's syndrome), it demonstrated an NPV of 99% and as such the model could be useful as a screening test early in the diagnostic pathway to rule out Cushing's syndrome as a likely explanation for the dog's clinical signs. Adrenal function tests and differentiating tests could then be pursued if Cushing's syndrome remains a probable diagnosis based on the tool's result, the dog's overall clinical picture and routine laboratory results. The study emphasizes ongoing validation efforts of this and other disease prediction tools are a worthwhile effort, when considering their use in populations with differing characteristics, such as in different countries or practice types.

## CONFLICT OF INTEREST DECLARATION

Authors declare no conflict of interest.

## OFF‐LABEL ANTIMICROBIAL DECLARATION

Authors declare no off‐label use of antimicrobials.

## INSTITUTIONAL ANIMAL CARE AND USE COMMITTEE (IACUC) OR OTHER APPROVAL DECLARATION

Authors declare no IACUC or other approval was needed.

## HUMAN ETHICS APPROVAL DECLARATION

Authors declare human ethics approval was not needed for this study.
